# Closing the treatment gap for dementia in India

**Published:** 2009-01

**Authors:** Amit Dias, Vikram Patel

**Affiliations:** Epidemiologist and Geriatrician, Coordinator 10/66 Dementia Research Group - India, Jt. Secretary, Alzheimer’s and Related Disorders Society of India, Department of Preventive and Social Medicine, Goa Medical College, Goa, India; 1 Professor of International Mental Health, London School of Hygiene and Tropical Medicine, UK and Sangath, Goa, India

**Keywords:** Dementia, treatment gap, community based interventions, primary care

## Abstract

There is a rich epidemiological evidence base on dementia in India which shows that this neurodegenerative condition is an important public health problem, particularly in the context of the rapid demographic transition in many parts of the country. Research has shown that most people with dementia, and their caregivers, have significant unmet health and social welfare needs. Due to the great shortage of health care resources and the low levels of awareness about dementia, interventions addressing the needs of the people should be home based and directed at improving quality of life of the person with dementia and the caregiver. In view of the lack of specialists to deal with dementia, a group in Goa developed an alternate model of care which involved training lay health workers to provide home-based care for people with dementia under the supervision of a psychiatrist. This was successfully implemented and evaluated in a randomized controlled trial which showed clear benefits. This article concludes by considering the implication of these findings on strategies for scaling up services and close the treatment gap for dementia in India.

## INTRODUCTION

Understanding the burden of dementia is vital for developing and promoting dementia services in the Low and Middle Income Countries (LMICs). Other articles in this special issue of the IJP provide a systematic overview of the epidemiology of dementia in India. In 2005, the 10/66 dementia research group, estimated that there were 24.3 million people with dementia in the world which increase at the rate of 4.6 million every year^1^. The study also estimated that the prevalence of dementia in India and South Asia was 1.9% in those ≥60 years with an annual incidence of 4.3/1000. The prevalence is estimated to reach 3.6 million by 2020 and 7.5 million by 2040 in this region[1]. The rate of increase was estimated to be 3-4 times higher in developing countries than in developed countries.[1]

There are a number of essential issues which need to be addressed in planning services for people with dementia in India, as listed below:

- What is the burden, in absolute numbers, of people affected?
- What are the help-seeking patterns of people with dementia?
- What is the estimated treatment gap for evidence based care?
- How should evidence based services be delivered, by whom and in what settings?

Each of these will be discussed in this article, concluding with implications of this evidence base for closing the treatment gap for dementia.

## THE BURDEN OF DEMENTIA IN INDIA

Since health is a state subject in India, it is important to estimate the burden of dementia in individual states of the country as a first step to planning services. Considering the prevalence of 1.9% cited earlier, we have estimated the numbers of persons with dementia in each states using the census 2001 data[2] as shown in Table 1. We estimate that there are, in total, nearly 1.5 million people living with dementia in India today. These numbers are expected to increase dramatically in the years ahead due to the demographic transition.

**Table 1:** Estimated cases of dementia in various states of India (based on census 2001)

State/ UT	Total population	% ≥60 yrs	Estimated people with dementia
Jammu and Kashmir	10143700	6.7	12913
Himachal Pradesh	6077900	9	10393
Punjab	24358999	9	41654
Chandigarh	900635	5	856
Uttaranchal	8485349	7.7	12414
Haryana	21144564	7.5	30131
Delhi	13850507	5.2	13684
Rajasthan	56507188	6.7	71934
Uttar Pradesh	166197921	7	221043
Bihar	82998509	6.6	104080
Sikkim	540851	5.4	555
Arunachal Pradesh	1097968	4.5	939
Nagaland	1990036	4.5	1701
Manipur	2166788	6.7	2758
Mizoram	888573	5.5	929
Tripura	3199203	7.3	4437
Meghalaya	2318822	4.6	2027
Assam	26655528	5.9	29881
West Bengal	80176197	7.1	108158
Jharkhand	26945829	5.9	30206
Orissa	36804660	8.3	58041
Chhattisgarh	20833803	7.2	28501
Madhya Pradesh	60348023	7.1	81409
Gujarat	50671017	6.9	66430
Daman and Diu	158204	5.1	153
Dadra Nagar Haveli	22490	4	17
Maharashtra	96878627	8.7	160140
Andhra Pradesh	76210007	7.6	110047
Karnataka	52850562	7.7	77320
Goa	1347668	8.3	2125
Lakshadweep	60650	6.1	70
Kerala	31841374	10.5	63523
Tamil Nadu	62405679	8.8	104342
Pondicherry	974345	8.3	1536
Andaman and Nicobar Islands	356152	4.9	332
Total	1454679		

## HELP-SEEKING FOR PEOPLE WITH DEMENTIA

For most people, the features characteristic of dementia are considered to be part of, or a non-pathological deviation from, normal ageing. A study in Goa looked at the attitudes towards mental health problems in elders amongst health care providers and family caregivers.[3] Although a vignette of dementia was widely recognized, the condition was not thought to constitute a health problem. Dementia was construed as a normal part of ageing and was not perceived as requiring medical care. Thus, primary health physicians rarely saw this condition in their clinical work, but community health workers frequently recognized individuals with dementia. Indeed, there was no label in the local language for dementia. Dementia was occasionally attributed to abuse, neglect, or lack of love on the part of children towards a parent. There was evidence that the system of family care and support for older persons was less reliable than has been claimed. Care was often conditional upon the child’s expectation of inheriting the parent’s property. Care for those with dependency needs was almost entirely family-based with little or no formal services. Not surprisingly, fear for the future, and in particular ‘dependency anxiety’ was commonplace among older persons. There is stigma associated with the psychological and behavioral problems associated with dementia. People are often neglected in their homes and sometimes abused.

Formal care arrangements for elders, for example in the public health sector, are scarce. The specialities of old age psychiatry or geriatric medicine are very poorly established in the country and there is virtually no concept of continuing care which meets the complex medical and psychosocial needs of people with dementia and their families.[4] The number of residential places for elders with severe mental disorders such as dementia is also very low; the most recent estimate we could find is nearly 10 years old and reported about 354 homes, more than half of which were in the two southern states of Kerala and Tamil Nadu. Thus, the family remains not only the primary source of care and support for the vast majority of elders in India, but in fact the only source as well.

Caring for people with dementia is associated with a greater physical, mental, and financial burden on the caregiver.[5] Studies by the 10/66 Dementia Research Network in Goa and Chennai examined the impact of care giving for elders. Carers of people with dementia spent significantly longer time providing care than did carers and co-residents of depressed persons and controls. The highest proportion of time was spent in communicating, supervising, and helping with eating and toileting. They were much more likely to have a common mental disorder than carers or co-residents of controls. Economic strain was indicated by the high proportion of caregivers of people with dementia who had given up work to provide care, coupled with the increased likelihood that the family had to meet relatively high health care costs. This was explained by the increased propensity for people with dementia to use expensive private medical care services rather than free or low cost government services.[5]

## THE TREATMENT GAP FOR DEMENTIA IN INDIA

The concept of the treatment gap describes the gap between the numbers of people with a health condition and the number of these people who receive at least basic evidence based care. The recent Disease Control Priorities Project identified the effective treatments for dementia.[6] Anticholinesterase drugs show benefits only for patients with mild to moderate dementia, and only in the short term. However, the high costs of these drugs mean that these are unlikely to be cost-effective in low resource environments. Training family caregivers in behavioral management techniques reduces the level of agitation and anxiety in people with dementia. Use of low doses of antipsychotic medications, which reduce symptoms such as aggression and wandering, have been shown to reduce caregiver stress. Interventions that have specifically targeted stress and depression among caregivers are also beneficial.

There are no accurate estimates for the treatment gap for dementia in India, but we estimate that this gap exceeds 90% in most parts of the country, with the exception of urban areas and the two southern states of Kerala and Tamil Nadu. We do have relatively accurate estimates of the treatment gap from Goa. In a recent study in which 81 subjects with dementia participated, although 41 (51%) were seen by a doctor in the previous three months, only 4 (5%) had received the diagnosis and treatment specific for dementia. Thus, the treatment gap was over 90%, even in this relatively prosperous state of India with relatively good public health and mental health services. The study also reported that some families refused to take dementia specific medications mainly citing reasons including high cost, the family doctor advising not to take the medications, and fear of side effects.[7]

There are several major barriers to closing this treatment gap including the low levels of awareness about dementia as a medical disorder; however, the most significant barrier is the very low human resource capacity for the care of people with dementia. This scarcity of resources is true for all mental disorders across the continuum of life and has been systematically documented in the recent Lancet series on Global Mental Health.[8] India, like most LMICs, lack economic and human capital to achieve widespread coverage of specialist services;[9] furthermore, specialist services tend to focus almost entirely on medical interventions, which have only a limited role in the long-term care of people with dementia. In view of the above, service development for families of people with dementia in India should keep in mind that the service should be home based, address the diverse medical and psychosocial health needs of the affected persons and their caregivers and be provided at a cost that the family can afford (therefore use public and low-cost service providers). Thus, the challenge for India is to develop culturally appropriate interventions that can be delivered within existing resources, such as supporting families in their role as caregivers. The model of care in developing countries should be based on home care, along with providing training and support for family caregivers.[6] Interventions that should not be pursued include the use of multiple medications, which can be detrimental in older age groups, particularly unproven medications such as cerebral activators and neurotropic agents.

## DELIVERING EVIDENCE BASED SERVICES AT A LOW COST: THE HOME CARE PROGRAM

A randomized controlled trial has recently been completed in two talukas of Goa evaluating the benefits of a low-cost, home-based intervention aimed at supporting families affected by dementia.[7] The intervention was delivered by a community team, one for each taluka. Each team comprised two full-time Home Care Advisors (HCA), and a part-time local psychiatrist from the public health services, and a part-time lay counselor (who was shared by both teams). The minimum requirements for being a HCA were knowledge of the local language, being literate, preferably passed higher secondary school, and motivated to be involved in the community care of older people. They received intensive training for a week through role-play and interactive training methods. The HCA were trained in key skills including listening and counseling skills, bereavement counseling, stress management, and health advice for common health problems. The specific components of the intervention carried out by the HCA were:

- Basic education about dementia (what is the disease, its course, its features, etc.).
- Education about common behavior problems and how they can be managed.
- Support to the caregiver, for example: for an elderly caregiver living alone with the patient, in activities of daily living.
- Referral to psychiatrists or the family doctor when behavior problems are severe and warrant medication intervention.
- Networking of families to enable the formation of support groups.
- Advice regarding existing government schemes for elders.

The HCA applied a flexible home-care program tailored to the needs of the individual and the family. The minimum frequency of visits was at least once a fortnight for six months. The maximum was based on the needs as assessed by the HCA. Thus, the visits could be more frequent depending on the need of that particular family. The HCA were supported, and supervised, by the two part-time specialists: two psychiatrists (one supporting each team) and one counselor (supporting both teams; the counselor was herself a carer for a parent with dementia). Each person with dementia was seen at least once by a local psychiatrist who confirmed the diagnosis of dementia and advised regarding use of medication for behavior and other common medical problems based on an agreed protocol. The caregiver and the person with dementia were encouraged to visit the psychiatrist in the clinic so that, if medication or clinical investigations were needed, these could be availed of at no cost from the public health service, and because the time required for travel for the psychiatrist for home visits was considered to be wasting a precious resource. A home visit was arranged if a clinic visit was not possible. HCA would meet the psychiatrist twice a month and update them on the progress of the person with dementia, particularly those who were receiving medication. The other specialist was a lay counselor who had herself been a caregiver for a parent with dementia. The HCA from both talukas met with the counselor once a fortnight to share experiences, support one another, and solve difficult situations. Thus, the intervention followed a flexible stepped care model aimed at improving the knowledge of the family caregivers, providing emotional support, maximize caregiving resources and improving caregiving skills. The intervention demonstrated a significant impact in reducing the caregiver burden, mental stress, and distress due to the behavioral and psychological symptoms of dementia. It also showed a non-significant reduction in the total number of deaths in people with dementia in the intervention arm.

## IMPLICATIONS FOR SCALING UP SERVICES FOR DEMENTIA IN INDIA

The Kyoto Declaration of the Alzheimer’s Disease International (ADI) identifies the minimal actions required for dementia care in low, medium, and high resource settings.[10] This was based on the recommendations by the WHO 2001[11] and is summarized in Table 2. The focus has been to integrate dementia services with the primary health care system. The Goa Home Care Program demonstrates that it is possible to introduce a community dementia outreach programme as part of the existing primary health care set up, relying on trained low-cost community health workers supervised by mental health or other appropriately trained specialists. We believe that it would not be ideal to involve the existing community health workers, such as multi- purpose workers for dementia care as they are already overburdened with programmes involving vector borne diseases, tuberculosis, reproductive and child health, immunizations, etc. However, there are a number of conditions which, like dementia, produce chronic disabilities (such as strokes) and which may benefit from community based psychosocial interventions. Thus, it is possible that the model of home based support we have developed may be extended to such conditions to create a ‘chronic disease counselor’ whose primary role is to support persons (and families) affected by chronic, disabling, conditions. Strengthening the community based interventions by training community workers under supervision in line with what is envisaged in the National Mental Health Programme which was launched in 1982.

We are also aware that the trial on which this intervention is based is a relatively small pilot study; we strongly advocate for a multi-center trial which can evaluate the feasibility, acceptability and effectiveness of this intervention in diverse settings in the country. Future research should also examine the possibility of replacing the psychiatrist (a scarce resource in many parts of India) by the general practitioner trained in dementia care. Several National Health programmes like the Revised National Tuberculosis Control programme are testimony to the fact that the medical doctor involved in the primary health care can be trained to manage serious illnesses affecting the community.

**Table 2:** Minimal actions required for dementia care (based World Health Report - 2001)

Recommendation	Scenario 1: Low level of resources	Scenario 2: Medium level of resources	Scenario 3: High level of resources
1. Provide treatment in primary care	Recognize dementia care as a component of primary health care Include the identification and treatment of dementia in training curricula of all health personnel	Develop locally relevant training materials Provide refresher training to primary care physicians (100% coverage in 5 years)	Improve effectiveness of management of dementia in primary health care
	Provide refresher training to primary care physicians (at least 50% coverage in 5 years).		Improve referral patterns
2. Provide community care	Establish the principle that people with dementia are best assessed and treated in their own homes	Initiate pilot projects on integration of dementia care with general health care	Develop alternative residential facilities Provide community care facilities (100% coverage)
	Develop and promote standard needs assessments for use in primary and secondary care	Provide community care facilities (at least 50% coverage with multidisciplinary community teams, day care, respite and inpatient units for acute assessment and treatment)	Give individualized care in the community to people with dementia
	Initiate pilot projects on development of multidisciplinary community care teams, day care and short term respite Move people with dementia out of inappropriate institutional settings	According to need, encourage the development of residential and nursing home facilities, including regulatory framework and system for staff training and accreditation	
3. Make treatment for dementia available	Increase availability of essential drugs for the treatment of dementia and associated psychological and behavioral symptoms	Ensure availability of essential drugs in all health care settings	Provide easier access to newer drugs (e.g., anticholinesterase agents) under public or private treatment plans
	Develop and evaluate basic educational and training interventions for caregivers	Make effective caregiver interventions generally available	
4. Promote mental health and fight stigma	Promote public awareness campaigns which will help fight stigma and discrimination	Use the mass media to promote awareness of dementia, foster positive attitudes, and help prevent cognitive impairment and dementia	Launch public campaigns for early help-seeking, recognition and appropriate management of dementia
	Support nongovernmental organizations in public education		
5. Involve communities, families and consumers	Support the formation of self-help groups Fund schemes for nongovernmental organizations	Ensure representation of communities, families, and consumers in policy-making, service development and implementation	Foster advocacy initiatives
6. Establish national policies, programmes and legislation	Revise legislation based on current knowledge and human rights considerations Formulate dementia care programmes and policies Legal framework to support and protect those with impaired mental capacity	Implement dementia care policies at national and subnational levels	Ensure fairness in access to primary and secondary health care services, and to social welfare programmes and benefits
	Inclusion of people with dementia in disability benefit schemes Inclusion of caregivers in compensatory benefit schemes Establish health and social care budgets for older persons	Increase the budget for mental health care	
7. Develop human resources	Train primary health care workers Initiate higher professional training programmes for doctors and nurses in old age psychiatry and medicine Develop training and resource centers	Create a network of national training centers for physicians, psychiatrists, nurses, psychologists, and social workers	Train specialists in advanced treatment skills
8. Partnerships with other sectors	Initiate community, school and workplace dementia awareness programmes	Strengthen community programmes	Occupational health services for people with early dementia
	Encourage the activities of nongovernmental organizations		Provide special facilities in the workplace for caregivers of people with dementia Initiate evidence-based mental health promotion programmes in collaboration with other sectors
9. Set up a surveillance and monitoring system for dementia	Include dementia in basic health information systems Survey high-risk population groups	Institute surveillance for early dementia in the community	Develop advanced monitoring systems Monitor effectiveness of preventive programmes
10. Support dementia research	Conduct studies in primary health care settings on the prevalence, course, outcome and impact of dementia in the community	Institute effectiveness and cost-effectiveness studies for community management of dementia	Extend research on the causes of dementia Carry out research on service delivery Investigate evidence on the prevention of dementia
